# Iron overload phenotypes and *HFE* genotypes in white hemochromatosis and iron overload screening study participants without *HFE* p.C282Y/p.C282Y

**DOI:** 10.1371/journal.pone.0271973

**Published:** 2022-07-27

**Authors:** James C. Barton, J. Clayborn Barton, Ronald T. Acton

**Affiliations:** 1 Department of Medicine, University of Alabama at Birmingham, Birmingham, Alabama, United States of America; 2 Southern Iron Disorders Center, Birmingham, Alabama, United States of America; 3 Department of Microbiology, University of Alabama at Birmingham, Birmingham, Alabama, United States of America; University of Copenhagen: Kobenhavns Universitet, DENMARK

## Abstract

**Background:**

Screening program participants with iron overload (IO) phenotypes without *HFE* p.C282Y/p.C282Y are incompletely characterized.

**Methods:**

We studied white participants who had IO phenotypes without p.C282Y/p.C282Y in post-screening clinical examinations (CE). We defined IO phenotypes as a) elevated serum ferritin (SF) and transferrin saturation (TS) at screening and CE, and b) absence of IO treatment, anemia, transfusion >10 units, alcohol intake >30 g/d, hepatitis B or C, and pregnancy. We defined IO-related disease as elevated alanine or aspartate aminotransferase (ALT/AST) or swelling/tenderness of 2nd/3rd metacarpophalangeal (MCP) joints. All participants had *HFE* p.C282Y and p.H63D genotyping.

**Results:**

There were 32 men and 26 women (mean age 54±16 y). Median food/supplemental iron intakes were 14.3/0.0 mg/d. Relative risks of *HFE* genotypes were 12.9 (p.C282Y/p.H63D), 3.0 (p.H63D/p.H63D), 1.9 (p.C282Y/wt), 0.9 (p.H63D/wt), and 0.5 (wt/wt) compared to 42,640 white screening participants without IO phenotypes or p.C282Y/p.C282Y. Regression on SF revealed positive associations: MCV (p = 0.0006; β coefficient = 0.4531); swelling/tenderness of MCP joints (p = 0.0033; β = 0.3455); and p.H63D/wt (p = 0.0015; β = 0.4146). IO-related disease (18 elevated ALT/AST, one swelling/tenderness of MCP joints) occurred in 19 participants (7 men, 12 women). Median MCV was higher in participants with IO-related disease (97 fL vs. 94 fL; p = 0.0007). Logistic regression on IO-related disease revealed a significant association with diabetes (p = 0.0416; odds ratio 18.9 (95% confidence interval 1.0, 341.1)).

**Conclusions:**

In the present 58 screening program participants who had IO phenotypes without *HFE* p.C282Y/p.C282Y, relative risks of *HFE* genotypes p.C282Y/p.H63D, p.H63D/p.H63D, and p.C282Y/wt were significantly higher than in 42,640 white screening participants with neither IO phenotypes nor p.C282Y/p.C282Y. SF was significantly associated with MCV, swelling/tenderness of 2nd/3rd MCP joints, and p.H63D/wt. IO-related disease was significantly associated with MCV and diabetes.

## Introduction

The *HFE* gene (homeostatic iron regulator, chromosome 6p23.1) [1] that encodes HFE, a negative upstream regulator of hepcidin [2], and two common non-ancestral *HFE* alleles p.C282Y (c.845G>A; rs1800562) and p.H63D (c.187C>G; rs1799545) were discovered in referred white patients with hemochromatosis and iron overload (IO) in 1996 [1]. This discovery stimulated population screening for hemochromatosis and IO based on a) measuring serum ferritin (SF), a surrogate marker of iron stores, and transferrin saturation (TS), a marker of iron absorption/transport and macrophage iron efflux, and b) detecting p.C282Y and p.H63D [3–5]. The combination of elevated SF and elevated TS detected in non-Hispanic whites who participate in hemochromatosis and IO population screening programs is often associated with hemochromatosis due to p.C282Y/p.C282Y [3–5].

Rare types of hemochromatosis, usually diagnosed in referred patients, are associated with novel *HFE* alleles [6] or deleterious alleles in genes that encode hemojuvelin BMP co-receptor (*HJV*, chromosome 1q21.1), transferrin receptor 2 (*TFR2*, chromosome 7q22.1), hepcidin antimicrobial peptide (*HAMP*, chromosome 19q13.12), and solute carrier family 40 member 1 (ferroportin) (*SLC40A1*, chromosome 2q32.2) [7]. Potential iron-related consequences of hemochromatosis, regardless of genetic type, include increased mean corpuscular volume (MCV), liver injury, arthropathy, diabetes, hypogonadotrophic hypogonadism, cardiomyopathy, and cirrhosis [7].

Some population screening program participants who have elevated SF and elevated TS without p.C282Y/p.C282Y also have IO phenotypes due to hemochromatosis, although these participants have been incompletely characterized. A primary report of the Hemochromatosis and Iron Overload Screening (HEIRS) Study displayed separate initial screening TS and SF data of participants from a racially diverse population segregated only for *HFE* genotype and sex [4]. Those displays do not identify subgroups of participants who had elevation of both screening TS and SF values and thus may have had hemochromatosis or IO phenotypes [4].

To learn more, we studied non-Hispanic whites who had IO phenotypes without *HFE* p.C282Y/p.C282Y who participated in the HEIRS Study post-screening clinical examinations (CE) [4, 8, 9]. Genotyping was limited to detection of p.C282Y and p.H63D [4, 8, 9]. We defined IO phenotypes as a) elevated serum ferritin (SF) and elevated transferrin saturation (TS) in both screening and CE, and b) absence of IO treatment, anemia, transfusion >10 units, alcohol intake >30 g/d, hepatitis B or C, and pregnancy. We defined IO-related disease as elevated alanine aminotransferase (ALT) or aspartate aminotransferase (AST) levels or swelling/tenderness of the 2nd/3rd metacarpophalangeal (MCP) joints [5]. We computed proportions and relative risks of *HFE* genotypes other than p.C282Y/p.C282Y and identified variables significantly associated with IO phenotypes and IO-related disease. We discuss our observations in the context of previous reports.

## Materials and methods

### Ethics statement

The National Heart, Lung, and Blood Institute/National Human Genome Research Institute HEIRS Study evaluated the prevalence, genetic, and environmental determinants, and potential clinical, personal, and societal impacts of hemochromatosis and IO in a multiethnic, primary care-based sample of 101,168 adults enrolled during the interval 2001–2003 at four Field Centers in the US and one in Canada [4]. The Study was conducted in accordance with the principles of the Declaration of Helsinki. The local Institutional Review Boards of the HEIRS Study Coordinating Center, Central Laboratory, and each Field Center approved the Study protocol that is described in detail elsewhere [8]. The Field Centers recruited volunteer participants ≥25 years of age who gave written informed consent [4, 8].

### Screening

The HEIRS Study recruited participants from a health maintenance organization, diagnostic blood collection centers, and public and private primary care offices in ambulatory clinics associated with five Field Centers [4]. Ninety-eight percent of self-reported non-Hispanic whites were recruited at Field Centers in Alabama, California, Ontario, and Oregon/Hawaii [10]. Screening included SF and TS measurements and *HFE* p.C282Y and p.H63D allele-specific genotyping [4].

### Clinical examinations

Invitations to participate in a post-screening CE were extended to all *HFE* p.C282Y homozygotes (regardless of screening SF and TS), to all participants whose screening SF and TS exceeded study thresholds, regardless of *HFE* genotype, and to selected participants who had normal screening SF and TS and *HFE* wt/wt, defined as absence of p.C282Y and p.H63D. Median interval between screening and CE participation was 8 months [11].

At CE, eligible participants were informed of their screening SF, TS, and *HFE* genotype. Each participant completed a questionnaire addressing medical history and medications [11] and a University of Hawaii Multi-Ethnic Dietary Questionnaire which provided estimates of daily dietary and supplemental iron and alcohol intake [12]. An HEIRS Study physician performed a focused physical examination on each participant [11].

### Clinical examination laboratory methods and reference ranges

All testing was performed at the HEIRS Study Central Laboratory (Fairview-University Medical Center Clinical Laboratory, University of Minnesota, Fairview, MN, USA). At CE, a morning blood sample was obtained after an overnight fast to measure SF and TS (Hitachi 9/11 Analyzer, Roche Applied Science, Indianapolis, IN, USA), to confirm *HFE* genotype based on p.C282Y and p.H63D allele-specific analyses [11], to measure complete blood counts (Beckman Coulter GenS, Beckman/Coulter, Fullerton, CA, USA), and to measure serum ALT, AST, and glucose (Hitachi 9/11 Analyzer, Roche Applied Science, Madison, WI, USA) [9]. Using control specimens that represented normal ranges of SF, the total coefficient of variation for the Hitachi 9/11 Analyzer was 5.82–6.78%. For higher range SF standards, the total coefficient of variation was 5.98–8.24% [9].

Elevated SF and TS were defined as SF >300 μg/L and TS >50% (men) and SF > 200 μg/L and TS >45% (women). Reference ranges for hemoglobin were 133–177 g/L (men) and 117–157 g/L (women) and for mean corpuscular volume (MCV) were 78–105 fL (men) and 78–106 fL (women) [13]. Serum ALT >41 IU/L (men) and >32 IU/L (women) were defined as elevated. Serum AST >38 IU/L (men) and AST >32 IU/L (women) were defined as elevated [11].

Reflex testing for hepatitis B surface antigen and hepatitis C antibody was performed in all participants with elevated ALT or AST (Vitros ECi, Ortho-Clinical Diagnostics Incorporated, USA). Participants with elevated ALT or AST and positivity for hepatitis B surface antigen were defined to have viral hepatitis B [9]. Participants with elevated ALT or AST and positivity for hepatitis C virus antibody were defined to have viral hepatitis C [9].

Obtaining liver specimens by biopsy, estimating liver iron content using imaging techniques, and performing therapeutic phlebotomy were beyond the scope of the HEIRS Study.

### Definitions of iron overload and iron overload-related disease

We defined IO phenotypes as the following: a) elevated SF and elevated TS at both screening and CE, and b) absence of IO treatment, anemia, erythrocyte transfusion >10 units, alcohol intake >30 g/d, hepatitis B or C, and pregnancy. We defined IO-related disease in participants with IO phenotypes according to modified criteria of Allen et al. [5]: elevated serum concentrations of ALT or AST or swelling/tenderness of the 2nd/3rd MCP joints.

### Participants in this study

We analyzed observations on all self-identified non-Hispanic white CE participants without *HFE* p.C282Y/p.C282Y who had IO phenotypes, who fasted overnight ≥8 h before CE, and whose data analyzed in this study were complete.

### Macrocytosis and microcytosis

Macrocytosis was defined as MCV greater than the respective sex-specific upper reference limits. Microcytosis was defined as MCV ≤77 fL.

### Iron and alcohol intake and body mass index

Estimates of dietary and supplemental iron and daily alcohol intake were taken from the Multi-Ethnic Dietary Questionnaire [12]. We defined abstainers as participants whose estimated daily alcohol intake was <0.1 g. Body mass index (BMI) was measured as kg/m^2^.

### Diabetes

Self-reported diabetes reported at screening was confirmed with medication reviews at CE [11]. We defined undiagnosed diabetes according to the criteria of the American Diabetes Association (CE blood glucose >126 mg/dL (6.99 mmol/L) after an overnight fast of ≥8 h) [14]. Participants with self-reported diabetes and others with undiagnosed diabetes were combined in the present diabetes classification.

### Swelling/Tenderness of 2nd/3rd metacarpophalangeal joints

Physicians who performed physical examinations at CE recorded these manifestations as present or absent [11].

### Statistical analyses

The dataset for analyses consisted of complete observations on 58 CE participants with IO phenotypes. Age, SF, TS, hemoglobin, and MCV are expressed to the nearest integer. Descriptive data are displayed as enumerations, percentages, proportions, means (± 1 SD), or medians (range). Analyses of continuous data using d’Agostino’s and Shapiro-Wilk tests revealed that age and BMI values were normally distributed and thus these values are displayed as mean ± 1 SD and were compared using Student’s t tests (two-tailed). Continuous variables that were not normally distributed are displayed as medians (range) and were compared using Mann-Whitney U tests (unpaired data) or Wilcoxon’s signed-ranks test (paired data). Percentages were compared using Fisher’s exact test (two-tailed). The significance of the difference between two independent proportions was calculated using the z-ratio and two-tailed values of p. We compared screening and CE SF and TS data using Pearson’s correlation coefficient.

We computed relative risks of *HFE* genotypes other than p.C282Y/p.C282Y. We performed forward stepwise regression on SF at CE using a) all independent variables available at CE (except TS) and b) the first independent variable with p ≥0.05 as the stopping rule. TS was not used as an independent variable because its positive correlation with SF at CE was significant. We display standardized coefficients β for each significant predictor variable in this regression.

We performed logistic regression on IO-related disease using all available independent variables (except TS, elevated ALT or AST, and swelling/tenderness of 2nd/3rd MCP joints) for which values of p in univariate comparisons were ≤0.1500. We calculated odds ratios (95% confidence intervals (CI)) for significant independent variables.

We defined values of p <0.05 to be significant. Analyses were performed with Excel 2000^®^ (Microsoft Corp., Redmond, WA, USA), GB-Stat^®^ (2003; Dynamic Microsystems, Inc., Silver Spring, MD, USA), and GraphPad Prism 8^®^ (2018; GraphPad Software, San Diego, CA, USA).

## Results

### Elevated SF and elevated TS in participants without *HFE* p.C282Y/p.C282Y

Screening observations in 43,453 non-Hispanic white participants (16,716 men, 26,737 women) were evaluable for SF and TS levels and *HFE* genotypes [10]. Both screening SF and screening TS were elevated in 1.4% (600/43,240) of non-Hispanic white participants without *HFE* p.C282Y/p.C282Y [10]. Elevated SF and elevated TS at both screening and CE occurred in 185 non-Hispanic white participants without p.C282Y/p.C282Y. Of these 185 participants, 58 (31.4%) met all inclusion criteria for the present study. At screening and CE, neither respective median values of SF nor median values of TS differed significantly in these 58 participants (Table 1).

**Table 1:** Elevated SF and elevated TS in 58 non-Hispanic white participants without *HFE* p.C28Y/ p.C282Y^a^.

Marker	Screening^b^	Clinical examination^c^	Value of p (Wilcoxon’s signed-ranks test)	Pearson’s correlation coefficient r; value of p
Median serum ferritin, μg/L (range)	426 (214, 5300)	400 (224, 5398)	0.9137	0.8697; <0.0001
Median transferrin saturation, % (range)	58 (46, 98)	60 (48, 58)	0.6358	0.2998; 0.0223

^a^ SF, serum ferritin; TS, transferrin saturation. By selection, participants had elevated SF and elevated TS at both screening and post-screening clinical examination (men >300 μg/L, women >200 μg/L) and elevated TS (men >50%, women >45%).

^b^ Pearson’s correlation coefficient of screening SF and TS was not significant (r = -0.0254; p = 0.8499).

^c^ Pearson’s correlation coefficient of clinical examination SF and TS was significant (r = 0.4113; p = 0.0013).

### *HFE* genotypes

**Fig 1:**
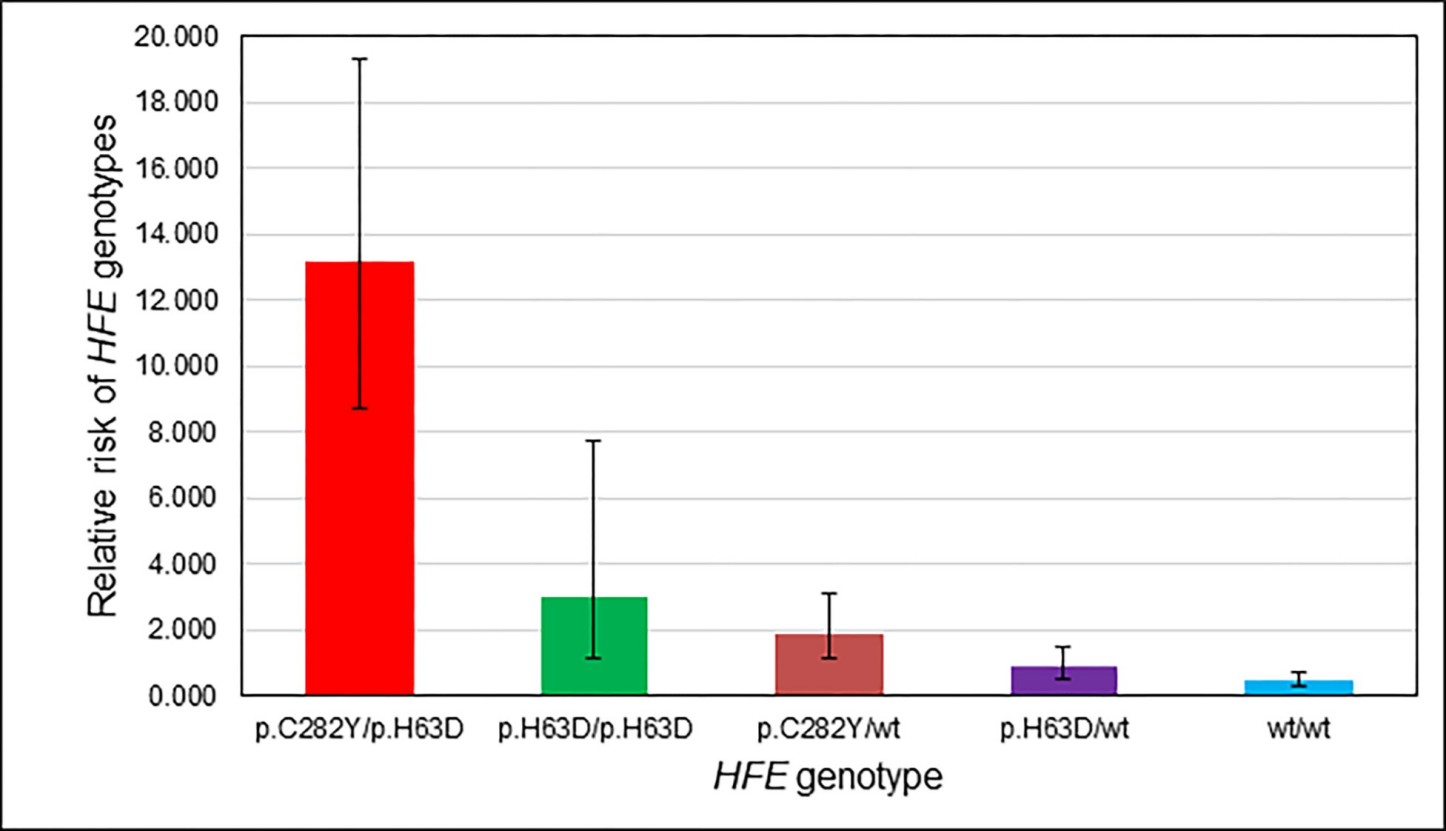
Relative risks (95% CI) of *HFE* genotypes in 58 non-Hispanic white post-screening clinical examination participants who had iron overload phenotypes without *HFE* p.C282Y/p.C282Y. Comparators were 42,640 non-Hispanic white HEIRS Study screening participants who had neither IO phenotypes nor p.C282Y/p.C282Y (see Table 3).

Percentages of *HFE* genotypes p.C282Y/p.H63D, p.H63D/p.H63D, and p.C282Y/wt in the present 58 CE participants with IO phenotypes were significantly greater than corresponding percentages in 42,640 non-Hispanic white screening participants without IO phenotypes (Table 2). Relative risks were 13.2 (p.C282Y/p.H63D), 3.0 (p.H63D/p.H63D), 1.9 (p.C282Y/wt), 0.9 (p.H63D/wt), and 0.5 (wt/wt) (Fig 1). Aggregate characteristics of participants with p.C282Y/p.H63D, p.H63D/p.H63D, or p.C282Y/wt and those of participants with p.H63D/wt or wt/wt did not differ significantly (S1 Table).

**Table 2:** *HFE* genotypes and iron overload phenotypes in non-Hispanic white participants without *HFE* p.C282Y/p.C282Y^a^.

*HFE* genotype	CE participants with IO phenotypes, % (n)^b^	Screening participants without IO phenotypes, % (n)^c^	z-ratio	Value of p^d^
p.C282Y/p.H63D, % (n)	31.0 (18/58)	1.8 (796/42,640)	16.233	0.0002
p.H63D/p.H63D, % (n)	6.9 (4/58)	2.2 (956/42,640)	3.273	0.0011
p.C282Y/wt, % (n)	20.7 (12/58)	10.0 (4,364/42,640)	2.624	0.0087
p.H63D/wt, % (n)	20.7 (12/58)	23.6 (10,248/42,640)	-0.596	0.5512
wt/wt, % (n)	20.7 (12/58)	60.5 (26,276/42,640)	-6.404	<0.0002

^a^ CE, post-screening clinical examination; IO, iron overload; SF, serum ferritin; TS, transferrin saturation; wt, absence of *HFE* p.C282Y and p.H63D.

^b^ IO phenotypes were defined as elevated SF (men >300 μg/L, women >200 μg/L) and elevated TS (men >50%, women >45%) at screening and CE.

^c^ 42,640 non-Hispanic white HEIRS Study screening participants had neither *HFE* p.C282Y/p.C282Y nor elevated SF and elevated TS at screening [10].

^d^ Differences between two independent proportions were calculated using z-ratios; values of p are two-tailed.

### 58 participants with iron overload phenotypes

Detailed characteristics of 58 participants are displayed in S2 Table. There were 32 men (55.2%) and 26 women (44.8%). Mean age was 54 ± 16 (SD) y. Mean ages of men (58 ± 17 y) and women (49 ± 17 y) did not differ significantly (p = 0.0719). Four of 26 women (15.4%; ages 30 y, 38 y, 38 y, and 40 y, respectively) reported that they were pre-menopausal.

Median MCV was 95 fL (77, 107). Macrocytosis was detected in one woman and microcytosis was detected in another woman. Median estimated daily iron intakes from food and iron supplements were 14.3 mg and 0.0 mg, respectively. Elevated ALT or AST occurred in 18 participants (31.0%). Swelling/tenderness of the 2nd/3rd MCP joints was observed in one participant (1.7%). Diabetes occurred in four participants (6.9%). *HFE* p.C282Y/p.H63D occurred in 18 participants (31.0%) and p.H63D/p.H63D in four participants (6.9%). Each of the genotypes p.C282Y/wt, p.H63D/wt, and wt/wt occurred in 12 participants. Two men and two women had SF >1000 μg/L (S3 Table).

Forward stepwise regression on SF at CE using other CE observations (except TS) as independent variables revealed three positive associations: MCV (p = 0.0006; β = 0.4531); swelling/tenderness of 2nd/3rd MCP joints (p = 0.0033; β = 0.3455); and *HFE* p.H63D/wt (p = 0.0015; β = 0.4146). This regression accounted for 32.0% of the variance of SF (regression ANOVA p = 0.0001).

### 19 participants with iron overload-related disease

These data are displayed in Table 3. Median MCV was higher in participants with than without IO-related disease (97 fL vs. 94 fL; p = 0.0007). Elevated ALT or AST or swelling/tenderness of 2nd/3rd MCP joints occurred only in participants with IO-related disease. Median estimated alcohol intake was significantly higher in participants with than without IO-related disease. Logistic regression on IO-related disease using independent variables with values of p ≤0.1500 (Table 3: male sex, MCV, median estimated alcohol intake, diabetes, and BMI) revealed one positive association: diabetes (p = 0.0416; odds ratio 18.9 (95% CI 1.0, 341.1)). This regression accounted for 28.4% of the deviance of IO-related disease (regression ANOVA p = 0.0009).

**Table 3:** Iron overload-related disease in 58 non-Hispanic white post-screening clinical examination participants^a^.

Characteristic	Iron overload-related disease (n = 19)	Iron overload phenotypes without iron overload-related disease (n = 39)	Value of p
Male, % (n)	36.8 (7)	64.1 (25)	0.0902
Mean age ± SD, y	55 ± 14	54 ± 17	0.8228
Median SF, μg/L (range)	398 (226, 875)	403 (224, 1218)	0.9934
Median TS, % (range)	62 (49, 85)	60 (48, 85)	0.7529
Median Hb, g/L (range)	146 (121, 175)	14.9 (120, 178)	0.2140
Median MCV, fL (range)	97 (89, 107)	94 (77, 103)	0.0007
Median estimated dietary iron intake, mg/d (range)	13.9 (2.9, 45.5)	0 (0, 63.5)	0.1665
Median estimated supplemental iron intake, mg/d (range)	0 (0, 13.5)	1.3 (0, 27.8)	0.5732
Median estimated alcohol intake, g/d (range)	6.2 (0, 30.0)	1.3 (0, 27.8)	0.0195
Diabetes	15.8 (3)	2.6 (1)	0.0982
Median BMI, kg/m^2^ (range)	28.1 (21.9, 40.7)	25.2 (21.1, 41.8)	0.0506
*HFE* p.C282Y/p.H63D, % (n)	31.6 (6)	30.8 (12)	1.0000
*HFE* p.H63D/p.H63D, % (n)	5.3 (1)	7.7 (3)	1.0000
*HFE* p.C282Y/wt, % (n)	15.8 (3)	23.1 (9)	0.7326
*HFE* p.H63D/wt, % (n)	21.0 (4)	20.5 (8)	1.0000
*HFE* wt/wt, % (n)	26.3 (5)	17.9 (7)	0.5023

^a^ ALT, alanine aminotransferase; AST, aspartate aminotransferase; BMI, body mass index; Hb, hemoglobin; MCP, metacarpophalangeal; MCV, mean corpuscular volume; SF, serum ferritin; TS, transferrin saturation; wt (wild-type), absence of *HFE* p.C282Y and p.H63D. Iron overload-related disease was defined as elevated ALT or AST or swelling/tenderness of 2nd/3rd MCP joints. Eighteen of 19 participants (94.7%) with iron overload-related disease had elevated ALT or AST. One of 19 participants (5.3%) with iron overload-related disease had swelling/tenderness of 2nd/3rd MCP joints.

^b^ Two of 14 women without iron overload-related disease (14.3%) and 2 of 12 women with iron overload-related disease (16.7%) reported that they were pre-menopausal (p = 1.0000).

### Relationships of MCV and alcohol intake

There were 15 abstainers (25.9%) and 43 non-abstainers (74.1%). The difference between median MCV values of abstainers and non-abstainers was not significant (94 fL (86–97) vs. 96 fL (77–107), respectively; p = 0.1369). All nine of 58 participants (15.5%) with MCV >100 fL were non-abstainers, although proportions of abstainers and non-abstainers with MCV >100 fL did not differ significantly (0% vs. 20.9%, respectively; p = 0.0941).

## Discussion

We evaluated observations in 58 non-Hispanic white participants in the HEIRS Study CE who had both elevated SF and elevated TS defined as IO phenotypes without *HFE* p.C282Y/p.C282Y who also reported that they had not received treatment for IO. Our study design excluded non-Hispanic white CE participants who may have had elevated SF and elevated TS due to types of anemia that increase iron absorption, a history of erythrocyte transfusion >10 units, viral hepatitis B or C, or increased alcohol intake. Elevated SF without elevated TS is common in patients with obesity [15], metabolic syndrome [16], diabetes [17], non-alcoholic fatty liver disease [18, 19], inflammatory disorders [16], malignancies [20–22], "classic" ferroportin hemochromatosis due to deleterious *SLC40A1* alleles [16], hereditary hyperferritinemia-cataract syndrome [16], and benign autosomal dominant hyperferritinemia [23]. Thus, our selection criteria also excluded participants who had elevated SF without elevated TS due to these or other conditions.

Percentages of *HFE* genotypes p.C282Y/p.H63D, p.H63D/p.H63D, and p.C282Y/wt in the present 58 CE participants with IO phenotypes were significantly greater than corresponding percentages in 42,640 non-Hispanic white screening participants without IO phenotypes. SF was significantly associated with MCV, swelling/tenderness of the 2nd/3rd MCP joints, and p.H63D/wt. IO-related disease was significantly associated with MCV and diabetes. IO and IO-related disease were not significantly associated with estimated intake of dietary or supplemental iron. Taken together, we infer that heritable factors in addition to common *HFE* genotypes other than p.C282Y/p.C282Y or acquired factors we did not study contribute to or account for IO phenotypes, IO-related disease, and other potential iron-related consequences of hemochromatosis in the present participants.

Relative risk of *HFE* p.C282Y/p.H63D was increased in the present participants, although we observed no significant association of IO-related disease with p.C282Y/p.H63D. Participants in an Australian population screening program with IO phenotypes and p.C282Y/p.H63D also had a low risk of IO-related disease (1/82 men, 0/95 women) [24]. In referred patients with p.C282Y/p.H63D, the prevalence of elevated SF and elevated TS was higher in those with non-alcoholic fatty liver disease, diabetes, or excessive alcohol intake [25]. In an analysis of 14 case-control studies of hemochromatosis, the pooled odds ratio of p.C282Y/p.H63D as a contributor to hemochromatosis IO was 32.0 (95% CI 18.5, 55.4) [26]. In a meta-analysis, there was a significant association of p.C282Y/p.H63D with provisional and documented IO [27].

Glyceronephosphate O-acyltransferase (*GNPAT*) p.D519G (rs11558492) occurred with greater prevalence in French and Australian subjects with *HFE* p.C282Y/p.H63D with higher SF than those with lower SF [28, 29]. The prevalence of cytochrome b reductase 1 (*CYBRD1*) promoter polymorphism rs884409 was significantly higher in French patients with p.C282Y/p.H63D with higher SF than those with lower SF [28]. In another study, SF and TS were higher in referred patients with p.C282Y/p.H63D who also had *HJV* p.N196K (c.588T>G; rs1020058448) or *HAMP* -72C>T [30]. In the same study, *HAMP* p.G71D (c.212G>A; rs104894696) did not modify SF and TS phenotypes [30]. Thus, non-*HFE* iron-associated alleles probably account in part for the variable penetrance of p.C282Y/p.H63D phenotypes.

Relative risk of *HFE* p.H63D/p.H63D was increased in the present participants, although we observed no significant association of IO-related disease with p.H63D/p.H63D, after adjustment for other variables. Proportions of the present participants and referred patients in hemochromatosis case series with p.H63D/p.H63D [1, 31–33] are low. One of the present participants with p.H63D/p.H63D had IO-related disease, consistent with single referred patients with p.H63D/p.H63D in two previous reports [1, 33]. In another report, each of three referred patients with p.H63D/p.H63D had IO [32]. In an analysis of 14 case-control studies of hemochromatosis, the pooled odds ratio of p.H63D/p.H63D as a contributor to hemochromatosis IO was 5.7 (95% CI 3.2, 10.1) [26]. In a meta-analysis, p.H63D/p.H63D was significantly associated with both provisional and documented IO [27]. Neither novel *HFE* alleles nor deleterious alleles in *TFR2*, *HAMP*, and *SCL40A1* were detected in referred hemochromatosis patients with p.H63D/p.H63D [34, 35].

Relative risk of *HFE* p.C282Y/wt was increased in the present participants, although we observed no significant association of IO-related disease with p.C282Y/wt. In an analysis of 14 case-control studies of hemochromatosis, the pooled odds ratio of p.C282Y/wt as a contributor to hemochromatosis IO was 4.1 (95% CI 2.9, 5.8) [26]. In a meta-analysis, p.C282Y/wt was weakly associated with IO including TS 45%-50% [27]. Compound heterozygosity of p.C282Y with p.S65C (c.193A>T; rs1800730), the third most common non-synonymous *HFE* coding region allele [6], occurred in some non-Hispanic white screening program participants [3, 36]. Referred patients with p.C282Y/p.S65C have mild IO phenotypes without IO-related disease [37, 38]. In two kinships, IO phenotypes but not IO-related disease were associated with double heterozygosity for p.C282Y and *HAMP* Met50del IVS2+1(-G) and *HAMP* p.G71D, respectively [39].

Relative risk of *HFE* p.H63D/wt was increased in the present participants. In an analysis of 14 case-control studies of hemochromatosis, the pooled odds ratio of p.H63D/wt as a contributor to hemochromatosis IO was 1.9 (95% CI 1.5, 2.5) [26]. Rarely, referred patients with p.H63D/wt and IO phenotypes or IO-related disease are discovered to be compound heterozygotes with novel *HFE* alleles, e.g., p.I105T (c.314T>C) [40], or with another novel *HFE* allele, e.g., p.E168Q (c.502G>C), in *cis* with p.H63D [41]. In a meta-analysis, there was no relation of provisional and documented IO with TS >55% in subjects with p.H63D/wt [27]. It is plausible but unproven that SF or TS levels are modified by a locus linked to p.H63D on chromosome 6p.

Estimated median daily intake of dietary iron in the present participants is similar to that in adults in the U.S. not selected for IO phenotypes [42]. SF in the present participants was not significantly associated with daily intake of either dietary or supplemental iron, consistent with HEIRS Study observations in CE participants with *HFE* p.C282Y/p.C282Y [43].

MCV was significantly associated with SF and IO-related disease in this study, after adjustment for other variables. In two previous reports, mean MCV values in referred hemochromatosis patients without *HFE* p.C282Y/p.C282Y were significantly higher than those of control subjects [44, 45]. Increased iron uptake and hemoglobin synthesis by immature erythroid cells account in part for higher values of MCV in referred hemochromatosis patients, regardless of IO-related disease or *HFE* genotype [44]. In one study, there was a dose-related association of chronic alcohol intake and MCV [46]. By design, we excluded participants with estimated daily alcohol intake >30 g. The median daily alcohol consumption of the present 58 participants was 3.3 g (24% of a standard 14-g alcoholic drink equivalent [47]) and that of 19 participants with IO-related disease was 6.2 g (44% of a standard 14-g alcoholic drink equivalent [47]). We observed that mean MCV values and proportions of participants with MCV >100 fL in abstainers and non-abstainers did not differ significantly. Macrocytosis, a surrogate marker of prolonged heavy alcohol intake [48] and many other conditions [49], occurred in only one of the present 58 participants. Thus, it is unlikely that alcohol intake accounts for the significant association of MCV with SF and IO-related disease in this study.

Four of the present 58 participants (4.9%; two men, two women) had SF >1000 μg/L. Their *HFE* genotypes were p.H63D (n = 2) and p.H63D/p.H63D and wt/wt (one each). In 20,306 Canadian HEIRS Study participants linked to the Ontario Death Registry 9 years after screening, all-cause survival was significantly decreased in the 34 participants with screening SF >1000 μg/L and *HFE* genotypes other than p.C282Y/p.C282Y (2 p.H63D/p.H63D, 15 p.H63D/wt or p.C282Y/wt, and 17 wt/wt) [50].

*HFE* genotypes other than p.C282Y/p.C282Y in adults with IO phenotypes have meaningful clinical correlates. Some adults with IO phenotypes without p.C282Y/p.C282Y have hepatic steatosis [32, 51] or chronic viral hepatitis [51] for which management in addition to therapeutic phlebotomy is indicated. Although many adults with IO phenotypes without p.C282Y/p.C282Y have less severe iron loading than some p.C282Y homozygotes [32, 52], others without p.C282Y/p.C282Y have severe IO [1, 32]. Adults without p.C282Y/p.C282Y who have severe IO phenotypes, like three of the present participants, may benefit from evaluation for non-iron liver disease, non-*HFE* hemochromatosis, or non-*HFE* "modifier" mutations. Guidelines for hemochromatosis management promulgated by expert groups in the U.S. [53] and Europe [54] recommend that all persons with hemochromatosis phenotypes undergo *HFE* genotyping. It is also recommended that first-degree relatives of persons diagnosed to have *HFE* hemochromatosis undergo TS/SF phenotyping and *HFE* genotyping [53, 54]. There is no evidence that detecting *HFE* alleles has disease-identification value other than to reveal IO risk.

A strength of this study is that our selection criteria included only participants with IO phenotypes who were likely to have hemochromatosis in the absence of *HFE* p.C282Y/p.C282Y and unlikely to have other disorders that sometimes present with IO phenotype mimics. Another strength is the availability of screening and CE data sufficient to demonstrate a) significant differences in proportions and relative risks of *HFE* genotypes other than p.C282Y/p.C282Y and b) significant associations of IO phenotypes and IO-related disease with other potential iron-related consequences of hemochromatosis. An uncertainty of this study is that we may have excluded participants with "classic" *SLC40A1* (ferroportin) hemochromatosis in whom SF but not TS is typically elevated [16].

In this study, we assessed *HFE* genotype risk associated with IO phenotype because participants invited to the CE (other than those with *HFE* p.C282Y/p.C282Y or wt/wt) were selected on the basis of having both screening SF and screening TS values above HEIRS Study thresholds, not on the basis of *HFE* genotype [8, 11]. The HEIRS Study did not measure liver iron content or perform therapeutic phlebotomy to quantify IO. Genotyping was limited to *HFE* p.C282Y and p.H63D. The HEIRS Study performed analyses to detect specific *HFE* alleles other than p.C282Y and p.H63D and specific *HJV*, *TFR2*, *HAMP*, *SLC40A1* alleles in a small proportion of screening participants [36]. None of those participants met criteria for inclusion in the present study. Regardless, evaluation and treatment of persons with IO phenotypes does not depend on genotype results [7].

## Conclusions

We conclude that proportions and relative risks of *HFE* genotypes p.C282Y/p.H63D, p.H63D/p.H63D, and p.C282Y/wt were increased in screening program participants who had IO phenotypes without p.C282Y/p.C282Y. SF was significantly associated with MCV, swelling/tenderness of 2nd/3rd MCP joints, and p.H63D/wt. IO-related disease was significantly associated with MCV and diabetes. Novel *HFE* alleles, deleterious alleles of *HJV*, *TFR2*, *HAMP*, or *SLC40A1*, iron-related "modifier" alleles, or acquired factors could contribute to IO phenotypes and IO-related disease in some of the present participants.

## Supporting information

S1 Table Characteristics of non-Hispanic white post-screening clinical examination participants with iron overload phenotypes. (PDF)

S2 Table 58 non-Hispanic white post-screening clinical examination participants with iron overload phenotypes. (PDF)

S3 Table Post-screening clinical examination participants with serum ferritin >1000 μg/L and elevated transferrin saturation without *HFE* p.C282Y/p.C282Y. (PDF)
