# Supplementary material for: Iron overload phenotypes and HFE genotypes in white hemochromatosis and iron overload screening study participants without HFE p.C282Y/p.C282Y
Source: PLoS One. 2022 Jul 27;17(7):e0271973. doi: 10.1371/journal.pone.0271973 (PMC9328571; doi:10.1371/journal.pone.0271973)
Supplement: S1 Table — (PDF) [file pone.0271973.s001.pdf]

S1 Table. Characteristics of non-Hispanic white post-screening clinical examination participants with iron overload phenotypes.[a]

Characteristic	*HFE* p.C282Y/p.H63D, p.H63D/p.H63D, or p.C282Y/wt (n = 34)	*HFE* p.H63D/wt or wt/wt (n = 24)	Value of p
Male, % (n)	52.9 (18)	58.3 (14)	0.7908
Mean age ± SD, y	56 ± 16	52 ± 16	0.3306
Median SF, µg/L (range)	416 (227, 2347)	388 (224, 5398)	0.5914
Median TS, % (range)	62 (51, 85)	57 (48, 85)	0.6547
Median Hb, g/L (range)	147 (121, 170)	151 (120, 178)	0.5431
Median MCV, fL (range)	96 (90, 107)	94 (77, 106)	0.1473
Median estimated dietary iron intake, mg/d (range)	14.5 (2.9, 45.5)	14.0 (6.6, 26.4)	0.9496
Median estimated supplemental iron intake, mg/d (range)	0 (0, 27.0)	3.9 (0, 63.5)	0.6181
Elevated ALT or AST, % (n)	29.4 (10)	33.3 (8)	0.7799
Median estimated alcohol intake, g/d (range)	2.0 (0, 29.4)	4.1 (0, 29.9)	0.7402
Diabetes, % (n)	5.9 (2)	8.3 (2)	1.0000
Median BMI, kg/m^2 (range)	27.3 (21.1, 41.8)	26.3 (21.8, 34.6)	0.6357
Swelling/tenderness of 2nd/3rd MCP joints, % (n)	0 (0)	4.2 (1)	0.4138

[a] ALT, alanine aminotransferase; AST, aspartate aminotransferase; Hb, hemoglobin; MCP, metacarpophalangeal; MCV, mean corpuscular volume; SF, serum ferritin; TS, transferrin saturation; wt (wild-type), absence of *HFE* p.C282Y and p.H63D.
