# Supplementary material for: Iron overload phenotypes and HFE genotypes in white hemochromatosis and iron overload screening study participants without HFE p.C282Y/p.C282Y
Source: PLoS One. 2022 Jul 27;17(7):e0271973. doi: 10.1371/journal.pone.0271973 (PMC9328571; doi:10.1371/journal.pone.0271973)
Supplement: S2 Table — (PDF) [file pone.0271973.s002.pdf]

S2 Table. 58 non-Hispanic white post-screening clinical examination participants with iron overload phenotypes.[a]

Characteristic	n
Male, % (n)	55.2 (32)
Mean age ± SD, y	54 ± 16
Median SF, µg/L (range)	400 (224, 875)
Median TS, % (range)	60 (48, 85)
Median Hb, g/L (range)	148 (120, 178)
Median MCV, fL (range)	95 (77, 107)
Median estimated dietary iron intake, mg/d (range)[c]	14.3 (2.9, 45.5)
Median estimated supplemental iron intake, mg/d (range)	0 (0, 63.5)
Elevated ALT or AST, % (n)	31.0 (18)
Median estimated alcohol intake, g/d (range)	3.3 (0, 30.0)
Diabetes, % (n)	6.9 (4)
Mean BMI ± kg/m^2	27.6 ± 4.6
Swelling/tenderness of 2nd/3rd MCP joints, % (n)	1.9 (1)
HFE p.C282Y/p.H63D, % (n)	31.0 (18)
HFE p.H63D/p.H63D, % (n)	6.9 (4)
HFE p.C282Y/wt, % (n)	20.7 (12)
HFE p.H63D/wt, % (n)	20.7 (12)
HFE wt/wt, % (n)	20.7 (12)

[a] ALT, alanine aminotransferase; AST, aspartate aminotransferase; Hb, hemoglobin; MCP, metacarpophalangeal; MCV, mean corpuscular volume; SF, serum ferritin; TS, transferrin saturation; wt (wild-type), absence of *HFE* p.C282Y and p.H63D.
