# Supplementary material for: Iron overload phenotypes and HFE genotypes in white hemochromatosis and iron overload screening study participants without HFE p.C282Y/p.C282Y
Source: PLoS One. 2022 Jul 27;17(7):e0271973. doi: 10.1371/journal.pone.0271973 (PMC9328571; doi:10.1371/journal.pone.0271973)
Supplement: S3 Table — (PDF) [file pone.0271973.s003.pdf]

S3 Table. Post-screening clinical examination participants with serum ferritin >1000 µg/L and elevated transferrin saturation without *HFE* p.C282Y/p.C282Y.[a]

Participant	1	2	3	4
Sex	M	M	F	F
Age, y	35	51	64	68
Serum ferritin, µg/L	2347	1218	2405	5398
Transferrin saturation, %	73	61	85	75
Hemoglobin, g/L	155	152	143	127
Mean corpuscular volume, fL	102	88	95	106
Estimated dietary iron intake, mg/d	13.9	9.6	14.7	9.5
Estimated supplemental iron intake, mg/d	0	0	0	3.9
Elevated ALT or AST	yes	no	no	yes
Estimated alcohol intake, g/d	1.1	20.3	0.006	29.9
Diabetes	yes	no	no	no
Body mass index, kg/m^2	40.7	25.1	27.6	28.1
Swelling/tenderness of 2nd/3rd MCP joints, % (n)	no	no	yes	no
HFE genotype	p.H63D/p.H63D	p.H63D/wt	wt/wt	p.H63D/wt

[a] ALT, alanine aminotransferase; AST, aspartate aminotransferase; MCP, metacarpophalangeal; wt (wild-type), absence of *HFE* p.C282Y and p.H63D.
